# A Sustainable Cold-Recycled Solution for the Surface Finishing of Unpaved Rural Roads

**DOI:** 10.3390/ma15113920

**Published:** 2022-05-31

**Authors:** Leonardo Urbano, Davide Dalmazzo, Pier Paolo Riviera, Orazio Baglieri, Ezio Santagata

**Affiliations:** Department of Environment, Land and Infrastructure Engineering, Politecnico di Torino, 10129 Torino, Italy; leonardo.urbano@polito.it (L.U.); pierpaolo.riviera@polito.it (P.P.R.); orazio.baglieri@polito.it (O.B.); ezio.santagata@polito.it (E.S.)

**Keywords:** reclaimed asphalt, mineral sludge, sustainability, unpaved roads, rural roads, cold-recycled mixtures, bitumen emulsion

## Abstract

This paper presents the results of an experimental investigation which was carried out with the purpose of assessing the performance-related properties of an emulsion-based cold-recycled mixture to be employed as a sustainable solution for the surface finishing of unpaved rural roads. This mixture contained significant quantities of recycled components (reclaimed asphalt and mineral sludge), and its composition was fine-tuned by following an innovative mix design procedure. Properties of these mixtures were studied by means of laboratory tests which considered key parameters, such as flowability, indirect tensile stiffness modulus, indirect tensile strength, moisture susceptibility and resistance to permanent deformation. It was found that, by means of the proposed mix design procedure, optimal dosages of the recycled components can be identified, thereby ensuring the achievement of the desired properties in terms of high workability and adequate stiffness and strength.

## 1. Introduction

Construction and maintenance operations of road pavements, both of primary and secondary infrastructures, require large volumes of selected granular materials, which entails the excavation and use of considerable quantities of natural resources [1]. The growing concerns related to environmental impacts and to climate change have led the international pavement construction community to move towards increasingly sustainable solutions, in which the depletion of non-renewable resources (virgin aggregates and binders of petroleum origin) is significantly reduced. In such a context, the use of the so-called alternative aggregates, such as waste materials and by-products, represents one of the most sustainable strategies adopted to meet current needs [2,3,4].

Reclaimed asphalt (RA), which comes from the milling of existing asphalt pavements and is formed by aggregate clusters coated by aged bituminous binders, is widely used as an alternative component in the construction of flexible pavements [5,6,7,8,9,10,11,12,13], since it is available in large quantities (in 2019 more than 48 million of tons of RA was available in the EU [14]).

One of the most cost-effective and environmental-friendly modes of employment of RA is represented by cold-recycling, in which RA is combined with asphalt emulsion or foamed bitumen to create a stable and durable paving mixture [15]. Although cold-recycling techniques have traditionally been used in low-volume roads due to the poorer mechanical properties of corresponding mixtures [8,16,17,18,19,20], promising research has been reported in which cold-recycled asphalt mixtures exhibited performance in the field comparable to that of standard bituminous layers containing exclusively virgin materials [21,22,23,24,25,26,27,28,29,30,31,32,33,34].

Mineral sludge (MS), obtained by the industrial washing to which aggregates are subjected in crushing plants, may also be considered as a possible alternative material to be used in pavement construction [35]. This is due to the fact that large amounts of sludge, constituted by fine mineral materials, are unavoidably produced during aggregate production, and it has been observed that their stockpiling and/or disposal is one of the major problems faced by the aggregate industry [36]. Several attempts have been made to reuse MS, especially in pavement subbase and base layers [37,38,39,40,41,42,43,44], and in the pavement foundations in tunnels [35,45,46,47,48], thereby confirming the possibility to turn this waste material into a valuable resource.

Most of the studies presented in the literature are focused on the reuse of waste materials and by-products in major infrastructures, although secondary roads, including those constituting the rural road network, may represent a more fruitful and cost-effective application for recycling processes [49].

The majority of the Italian rural road network is constituted by unpaved gravel roads, referred to as low-type surfaces due to the low traffic volumes to which they are normally subjected [50]. Their basic structure consists of a gravel layer, laid on the existing natural soil, whose thickness depends on several factors, such as predicted traffic volume and the bearing capacity of the supporting subgrade.

Despite the low traffic volumes to which they are subjected, rural roads may be affected by several distresses which compromise their serviceability. In particular, they are especially sensitive to water drainage which is a key factor in controlling long-term durability through the prevention of surface erosion and saturation of the subgrade. Since maintenance operations may be quite frequent for the restoration of surface evenness and for the repair of localized potholes and ruts, in some cases it could be appropriate, although not necessarily cost-effective, to proceed with the application of a surface finishing bituminous-bound protective layer [51].

In this paper, a sustainable and cost-effective solution for the surface finishing of unpaved rural roads is proposed. An emulsion-based cold-recycled mixture (CRM) containing large quantities of RA and MS was conceived of and thereafter fine-tuned by following an innovative mix design procedure. Optimal composition was identified with the purpose of guaranteeing high workability (to reduce compaction efforts) and adequate stiffness and strength (to limit the frequency of maintenance operations). In order to maximize environmental benefits and to reduce the economic impact of the suggested solution, it was considered appropriate to avoid the use of any hydraulic binder (such as Portland cement) [52].

Workability was assessed by performing flowability tests on loose mixtures and by analyzing compaction curves obtained by making use of the gyratory shear compactor. Compacted specimens of the investigated CRMs were then subjected to indirect tensile stiffness modulus tests and to indirect tensile strength tests, and moisture susceptibility was evaluated by referring to the indirect tensile strength ratio. Additional tests were carried out on laboratory-compacted slabs of the optimal CRMs for the assessment of their resistance to permanent deformation.

## 2. Materials and Test Methods

### 2.1. Constituent Materials

**Figure 1:**
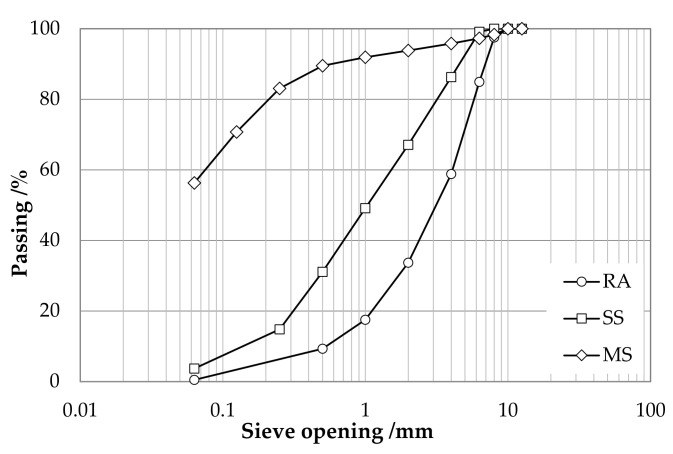
Particle size distribution of reclaimed asphalt (RA), sand (SS) and mineral sludge (MS).

Components employed for the formation of the aggregate skeleton of the investigated cold-recycled mixtures (CRMs) were reclaimed asphalt (RA, 0–12.5 mm), siliceous sand (SS, 0–6 mm) and mineral sludge (MS). All components were subjected to the preliminary determination of particle size distribution and apparent density (ρ_a_) as per the corresponding EN standards [53,54]. Results are shown in Figure 1 and Table 1.

The RA material employed in the investigation, sampled from a production plant where it had been subjected to preliminary sieving to discard particles larger than 12.5 mm, was derived from the milling of aged, dense-graded asphalt mixtures. Its binder content, which was determined as per EN 12697-39 [55], was found to be equal to 5.7% by weight of aggregates. As a consequence of its highly aged conditions, bitumen contained in the RA was considered as an inert component in the cold-recycled mixture; no diffusion phenomena occurred when in contact with the bitumen residue coming from the bituminous emulsion used in the mixtures.

The MS was collected from stockpiles of an aggregate crushing plant, and it was originated by the normal operation of washing to which aggregates are subjected in their production process.

The bituminous emulsion used for the preparation of the mixtures was C55B3 type (according to EN 13808 [56]) and had a binder content of 47%. The main characteristics of the residual bitumen are listed in Table 2.

### 2.2. CRMs

The aim of achieving an acceptable level of workability of the investigated CRMs suggested the adoption of an approach which is similar to that normally referred to in the design of self-compacting cementitious mixtures [35,45,46,47,48,57]. Thus, dosages of RA, sand and mineral sludge were obtained by considering the target size distribution proposed by Funk and Dinger [58], given by Equation (1):

(1) $$P(D)=100\cdot \frac{D^{q}-D_{\min}^{q}}{D_{\max}^{q}-D_{\min}^{q}}$$

where D represents the diameter of aggregate particles (in mm), P(D)—expressed in %—is the cumulative percentage passing the sieve with opening equal to D; D_max_ is the maximum diameter of aggregate particles in the mixture (in mm, fixed at 12.5 and corresponding to a P(D) equal to 100%); D_min_ is the minimum diameter of aggregate particles in the mixture (in mm, assumed to be equal to 5 μm); and q is the so-called distribution modulus, which may vary between 0 and 1 and defines the balance of coarse and fine aggregates within the aggregate skeleton (as q decreases, the aggregate skeleton of the mixture becomes finer).

From a conceptual viewpoint, it was assumed that mechanical properties (such as stiffness and strength) are mainly attributable to the stone-to-stone contact occurring between the coarse RA particles bound by the bituminous residue of the emulsion (which provides some degree of shear resistance), and flowability properties are mainly controlled by MS and added water. Thus, several trial mixtures were prepared by changing the value of the distribution modulus q and the RA content.

**Figure 2:**
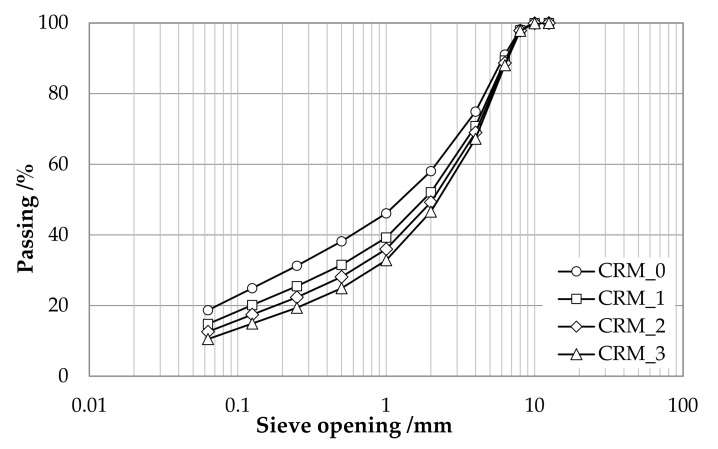
Particle size distribution of investigated CRMs.

The compositions of the aggregate skeletons of CRMs included in the present study are shown in Table 3 and Figure 2.

As mentioned in the Introduction, to ease laying and compaction procedures, special attention was paid to the workability features of the investigated mixtures.

Since the total fluid phase (%FP)—given by the contributions of bituminous emulsion (%E) and added water (%W)—lubricates particle-to-particle contacts, and therefore enhances the workability of CRMs [59], it was deemed appropriate to focus on the identification of its optimal value. Thus, as described further in this paper, flowability was evaluated by making use of the flow consistency test [60] typically used for the assessment of controlled low strength materials, which belong to the category of self-levelling and self-compacting cementitious mixtures.

Based on preliminary results obtained on trial CRMs, the dosage of the bituminous emulsion, expressed with respect to the total weight of the dry components of the lithic skeleton, was chosen to be 4% and kept constant throughout the experimental investigation.

All mixtures were manually prepared and mixed by adopting a protocol which entailed the following steps: (i) preliminary mixing of RA and bituminous emulsion to completely cover RA particles, (ii) addition of sludge, sand and water, (iii) continuous mixing of the resulting blend until the achievement of a homogeneous material.

The time elapsed between mixing and compaction was carefully controlled and kept constant—30 min—during which, a clear transition of the color of the mixture from brown to black was observed, thereby revealing the initiation of the breaking process of the emulsion.

### 2.3. Test Methods

The experimental program included laboratory tests on loose mixtures for the evaluation of the flowability characteristics and volumetric and mechanical characterization tests carried out on laboratory-compacted specimens.

Flowability tests were performed according to ASTM D6103 [60]. As prescribed by the standard protocol, an open-ended cylinder of 75 mm diameter and 150 mm height was filled with the mixture, which was thereafter allowed to spread over a non-absorbent flat surface by lifting the cylinder. Each blend of aggregates corresponding to the four candidate mixtures listed in Table 3 was mixed with progressively increasing water content. Spread diameter (Ds) was then measured, and the test sample was visually observed to identify the occurrence of any segregation or bleeding phenomena.

Cylindrical specimens (150 mm diameter) were prepared by making use of a gyratory shear compactor [61] with a fixed number of gyrations, equal to 100, at room temperature. The adopted compaction protocol differed substantially from the standard one used in the case of hot asphalt mixtures, since a bottom-perforated plate was used in order to allow the excess fluid phase to unidirectionally seep through the specimen [62]. During the compaction process, the height h_x_ of the specimen was recorded after each gyration to evaluate the progressive degree of compaction C_x_ (which represents the complement to 100 times the void content of the sample) by making use of Equation (2):

(2) $$C_{x}=100\cdot \frac{\rho_{\mathrm{geo},x}}{\rho_{m}}$$

where ρ_geo,x_ represents the geometric density of the sample at the generic gyration x and ρ_m_ is the maximum density measured on loose blends with the pycnometer method. ρ_geo,x_ was calculated as the ratio between the mass of the sample and the volume of a cylinder of 150 mm diameter and height h_x_ [63,64]. Densification curves were therefore obtained by analyzing the variation of C_x_ as a function of the number of gyrations recorded during the compaction process. For each mixture, a total of eight specimens were compacted. Volumetric properties were then calculated in terms of geometrical void content [65].

After compaction, in order to mimic long-term conditions reached in the field, test specimens were subjected to a curing procedure in a forced draft oven kept at 40 °C for 72 h.

Compacted specimens were then divided into two subsets, with approximately the same average height and average bulk density, named “dry subset” and “wet subset”. The dry subset was subjected to the assessment of stiffness modulus and indirect tensile strength, and the wet subset was conditioned in water for the evaluation of indirect tensile strength ratio.

According to EN 12697-26 Annex C [66], stiffness moduli were measured at 20 °C in the indirect tensile configuration, a non-destructive method in which cylindrical specimens are subjected to a repeated pulse loading (with a 125 ms rise time and 7 μm target horizontal elongation). The measured stiffness modulus was determined using the following formula (Equation (3)):

(3) $$E=\frac{F\cdot \left(\nu +0.27\right)}{\left(z\cdot h\right)}$$

where F is the peak value of the applied vertical load, z is the peak total horizontal elongation caused by loading, h is the mean thickness of the cylindrical specimen and ν is Poisson’s ratio (typically assumed equal to 0.35 for bitumen-bound materials at 20 °C).

Indirect tensile strength (ITS) was determined according to EN 12697-23 [67] at a displacement rate of 50.8 mm/min and at 20 °C on both subsets for the measurement of ITS_dry_ and ITS_wet_. The wet-subset was preventively soaked in a water bath at 20 °C for 24 h before testing [68].

ITS and indirect tensile strength ratio (ITSR) were calculated as follows (Equations (4) and (5)):

(4) $${\mathrm{ITS}}_{\mathrm{dry},\mathrm{wet}}=\frac{2\cdot P}{\pi \cdot d\cdot h}$$

(5) $$\mathrm{ITSR}=\frac{{\mathrm{ITS}}_{\mathrm{wet}}}{{\mathrm{ITS}}_{\mathrm{dry}}}\cdot 100$$

where P is the peak value of the vertical load; d and h represent the mean diameter and thickness of the cylindrical specimen, respectively.

In the case of the CRMs identified as optimal, slabs (50 cm length, 18 cm width, 5 cm thickness) were prepared by means of a large-size roller compactor, operating according to EN 12697-33 [69], with the same geometric air void content obtained in gyratory compaction.

On compacted slabs, the evaluation of the resistance to permanent deformation (also referred to as rutting resistance) was performed. Thus, wheel tracking tests were performed in accordance with EN 12697-22 [70]. The adopted protocol required the initial application of 1000 loads at room temperature in order to eliminate any surface defects and to establish the zero-point condition for rut measurements. Permanent vertical deformations were measured in 27 different points on the surface of each slab, at a temperature value of 20 °C, after 1000, 3000, 10,000 and 30,000 load applications, applied with a rubber wheel. The proportional rut depth was calculated using Equation (6):

(6) $$P_{i}=\overset{27}{\underset{j=1}{\sum }}\frac{\left(m_{\mathrm{ij}}-m_{0j}\right)}{27\cdot h}$$

where P_i_ is the measured proportional rut depth on the i-th load application, m_ij_ is the local deformation at the jth location after application of the i-th load, m_0j_ is the zero-point condition and h is the average initial slab thickness. Two replicates were carried out for each CRM and average values were considered in the analysis.

## 3. Results

### 3.1. Definition of the Fluid Phase

**Figure 3:**
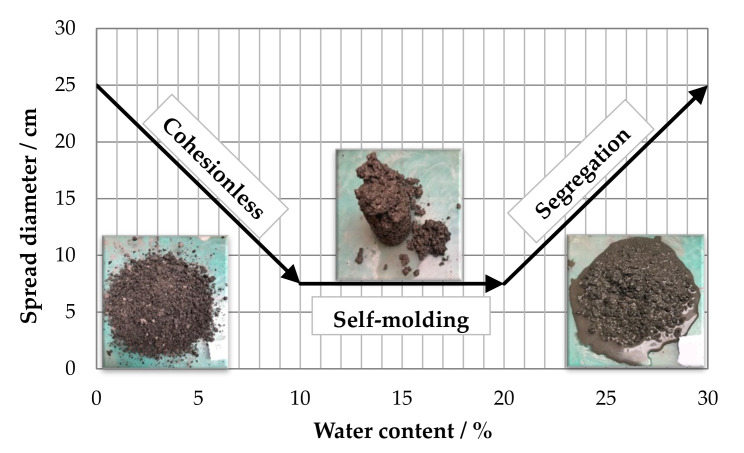
Flowability trend of CRMs.

Results obtained from flowability tests showed a particular behavior common to all investigated mixtures, as indicated in Figure 3. It can be observed that as the water content increased, three different regions were recognized, indicated as “cohesionless”, “self-molding” and “segregation”.

For very low water dosages, all CRMs showed a behavior similar to that of unbound granular mixtures, characterized by the absence of any significant cohesion between aggregate particles. By gradually increasing the water content, apparent cohesion of the mixtures began to be mobilized, leading to a progressive reduction in the spread diameter until the transition to a different behavior, indicated as self-molding. In this condition, the mixtures reached a level of cohesion which was sufficient to maintain the cylindrical shape without any spreading. This behavior, graphically described by a diameter equal to the internal diameter of the spread cylinder, occurred as long as the water content did not induce segregation phenomena, which caused the uncontrolled increase in spreading.

Based on the observations provided above, the self-molding region was considered as a characteristic feature of the workability and compactability of CRMs, and its range of water contents was considered as the range of target fluid phase contents to be explored in the further phases of the investigation.

**Figure 4:**
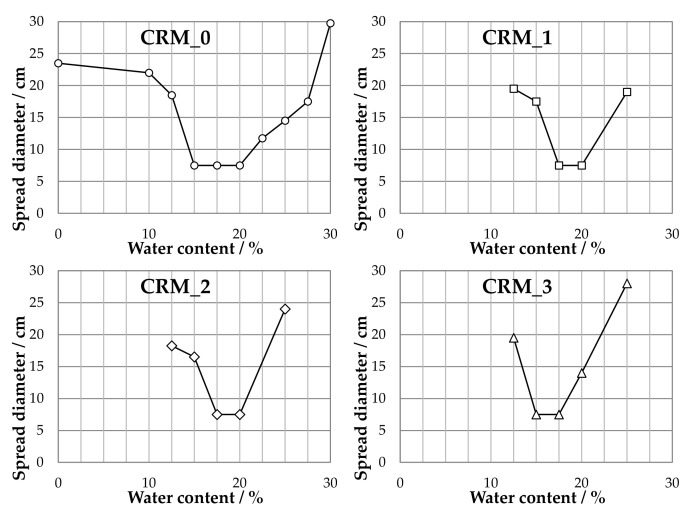
Results of flowability tests carried out on the CRMs.

Results of flowability tests obtained for the four considered CRMs are plotted in Figure 4 and synthesized in Table 4.

It can be observed that the self-molding regions of the investigated CRMs changed as functions of the compositions of their solid skeletons. In particular, it was found to be strongly dependent upon the amount of MS, which, based on its composition, can be considered as a water-absorbing fraction. The lower the MS dosage, the lower the amount of water required to achieve this behavior.

For each mixture, both water contents listed in Table 4 were employed as target values of the total fluid phase to be adopted in the preparation of CRMs subjected to volumetric and mechanical characterization. The resulting compositions of these mixtures are reported in Table 5, which highlight the contributions of the two components constituting the fluid phase (emulsion, set to 4%, and added water).

### 3.2. Densification Curves and Volumetric Properties

**Figure 5:**
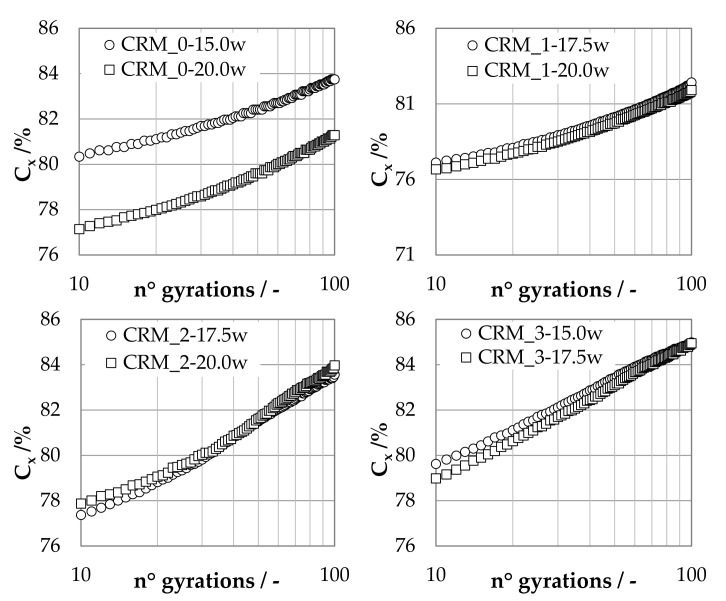
Average densification curves of investigated CRMs.

Average densification curves of investigated CRMs are summarized in Figure 5.

By analyzing the data plotted in Figure 5, it can be observed that the lowest degree of compaction was achieved by the CRM with the highest values of the total fluid phase percentage and fine content. Due to the greater volume of water absorbed by the MS, a limited drainage from the specimen was recorded during compaction, leading to a larger volume of internal air voids assessed after drying.

Moreover, it was noticed that differences between mixtures with the same aggregate skeleton but with different fluid phases were quite small in the case of CRM_1, CRM_2 and CRM_3, characterized by limited extension of the self-molding region (equal to 2.5%) and by greater amounts of RA particles. On the contrary, mixture CRM_0 revealed more pronounced variations in the densification curves due to the wider range of the self-molding region (equal to 5%) and a larger amount of fine particles.

**Figure 6:**
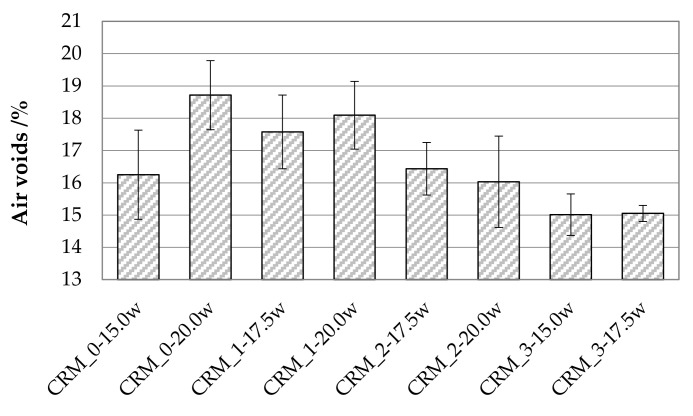
Average air void contents of investigated CRMs.

The volumetric properties of the compacted specimens, expressed in terms of geometric air void content, are presented in Figure 6, where the average values of each CRM are presented together with corresponding data dispersion bars.

Despite the high variability highlighted by the data dispersion bars, a characteristic trend could be defined as a function of the fluid phase percentage and of the solid skeleton composition. In particular, consistently with the observations made from the densification curves, the CRMs characterized by the highest values of the percentage of total fluid phase showed the highest void contents, and the reduction of MS content was found to lead to higher compaction levels.

### 3.3. Stiffness

**Figure 7:**
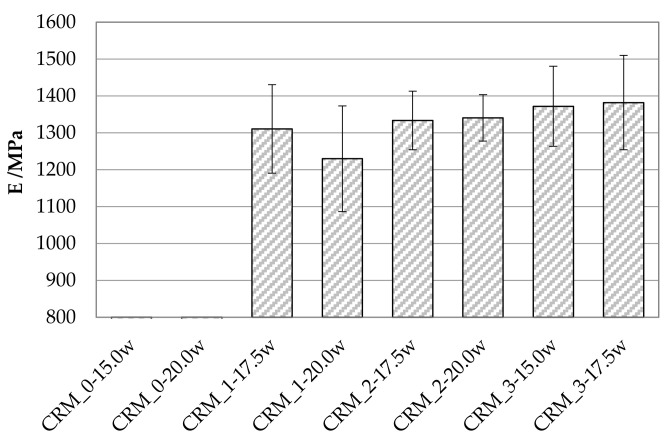
Stiffness moduli of investigated CRMs.

Stiffness moduli were measured at 20 °C on compacted specimens after curing. Average values obtained for each CRM are reported in Figure 7 together with the corresponding data dispersion bars.

Both CRM_0 mixtures failed during the application of conditioning load pulses, revealing poor mechanical behavior due to the weak stone-to-stone contact created between RA particles as a consequence of the higher dosages of the finer fractions (MS and SS). On the contrary, the increase in the RA fraction, together with the reductions in the MS and SS dosages, let to the creation of tougher internal structures with enhanced stiffness, of the order of 1300 MPa, of the same order of magnitude of more conventional cold-recycled mixtures [16]. As expected, the CRMs characterized by lower void content exhibited the higher values of stiffness modulus.

### 3.4. Indirect Tensile Strength (ITS) and Indirect Tensile Strength Ratio (ITSR)

**Figure 8:**
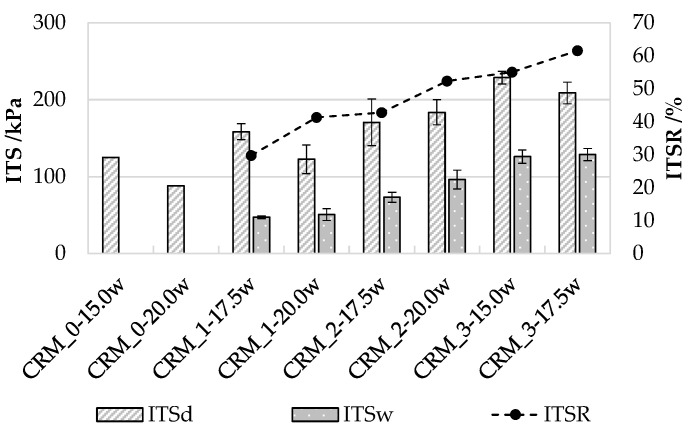
Indirect tensile strength (ITS_d_ in dry conditions, ITS_w_ after soaking) and strength ratio (ITSR) at 20 °C.

Results of indirect tensile strength tests, performed in dry (ITS_d_) and wet (ITS_w_) conditions, together with the corresponding indirect tensile strength ratio (ITSR) values, are reported in Figure 8. CRM_0 mixtures presented extremely low resistance after the conditioning in water, and their ITS_w_ values could not be determined.

Consistently with the outcomes of stiffness tests, in the case of indirect tensile strength it was found that the dosage increase in the coarser aggregate fraction (RA) provided improved stone-to-stone contact, thereby enhancing strength. The highest values of ITS, in both dry and wet conditions, were recorded for the CRM_3 mixtures, which were characterized by the highest RA dosage (75%) considered in the investigation. Both dry and wet ITS values showed that CRMs with lower void contents exhibited higher resistance to loading as a consequence of their denser structure.

ITSR results confirmed the enhanced mechanical properties provided by the combined effect of high RA dosage and low void content, which translated into improved water durability properties. However, it should be underlined that the obtained ITSR values were quite low, revealing drawbacks related to the moisture sensitivity of CRM mixtures, probably associated with their limited degree of compaction. This specific outcome of the investigation will be addressed in more detail in future investigations, before any full-scale implementation.

### 3.5. Rutting Resistance

Results gathered from volumetric and mechanical tests performed on the investigated CRMs led to considering the CRM_3 mixtures as the optimal ones, due to their high stiffness and strength.

Thus, further tests were performed on compacted slabs to assess resistance to permanent deformation. Slabs were compacted by means of a roller compactor to achieve the same geometric density of the corresponding gyratory-compacted specimens and were thereafter subjected to wheel tracking tests (performed at 20 °C).

**Figure 9:**
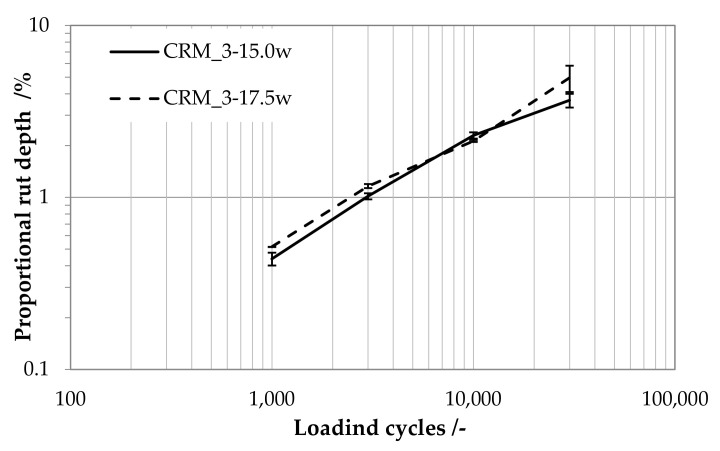
Proportional rut depth as a function of loading cycles at 20 °C.

The tendency of the CRMs to accumulate permanent deformation is graphically represented in Figure 9, in which the average values and data dispersion bars of the proportional rut depth (%) are plotted as a function of loading cycles.

Both CRMs showed similar and satisfactory anti-rutting behavior as a consequence of their high stiffness and low voids content. Visual inspection of the slabs at the end of the tests revealed the absence of cracks, raveling or particle loss, thereby confirming that mixtures of the considered type may constitute a valuable solution for the surface finishing of unpaved rural roads.

## 4. Conclusions

In this paper, it was shown that reclaimed asphalt (RA) and mineral sludge (MS) obtained from the production of aggregates can be successfully employed for the creation of cold-recycled mixtures (CRMs) to be employed as a sustainable solution for the surface finishing of unpaved rural roads. These mixtures are constituted by large quantities of recycled components (RA and MS) combined with small amounts of silica sand, bituminous emulsion and added water. Mix design was carried out according to an innovative procedure, by means of which optimal composition was identified by considering flowability, stiffness and strength.

The main findings of the study can be summarized as follows:

- From flowability tests, three different behaviors were observed as a function of the percentage of fluid phase, indicated as “cohesionless”, “self-molding” and “segregation”.
- The “self-molding” region was considered to represent the target condition to achieve in order to obtain CRMs characterized by satisfactory workability and compactability.
- High values of the percentage of fluid phase and of the MS dosage resulted in high volumes of voids, indicating that these composition parameters need to be carefully controlled in order to achieve a satisfactory compaction level.
- It was found that the mechanical properties of CRMs were mainly influenced by the RA content: its increase, together with reductions in the MS and SS dosages, led to the formation of tough internal structures with enhanced stiffness and strength.
- The CRM identified as optimal during the investigation showed a satisfactory performance in terms of resistance to permanent deformation.
- As a result of their limited degree of compaction, all CRMs exhibited significant moisture sensitivity.

Notwithstanding the fact that the obtained experimental results are considered to be extremely encouraging, further investigations will be carried out to refine the mix design methodology and to improve the performance properties of CRMs in terms of their moisture sensitivity. Field trials will be consequently planned to optimize the production and laying technology of these innovative mixtures, and to analyze their actual behavior when subjected to traffic loading.

## Figures and Tables

**Table 1:** Apparent density of reclaimed asphalt (RA), sand (SS) and mineral sludge (MS).

Fractions	ρ_a_ (kg/m^3^)
RA (0–12.5) mm	2562
SS (0–6) mm	2757
MS	2819

**Table 2:** Characteristics of bitumen contained in the emulsion (data provided by the supplier).

Characteristics	Unit	Value
Penetration @25 °C	0.01 mm	≤100
Softening point	°C	≥43
Fraass breaking point	°C	≤−10

**Table 3:** Composition of aggregate skeleton of investigated CRMs.

Mixture	q	RA	SS	MS
CRM_0	0.21	50%	17%	33%
CRM_1	0.30	65%	10%	25%
CRM_2	0.34	70%	9%	21%
CRM_3	0.38	75%	7%	18%

**Table 4:** Extreme limits of the self-molding region.

Mixture	Self-Molding Region (%)	
CRM_0	15.0	20.0
CRM_1	17.5	20.0
CRM_2	17.5	20.0
CRM_3	15.0	17.5

**Table 5:** Percentages of fluid phase, bituminous emulsion and added water of investigated CRMs.

Mixture	% FP	% E	% W
CRM_0_15.0w	15.0	4.0	11.0
CRM_0_20.0w	20.0	4.0	16.0
CRM_1_17.5w	17.5	4.0	13.5
CRM_1_20.0w	20.0	4.0	16.0
CRM_2_17.5w	17.5	4.0	13.5
CRM_2_20.0w	20.0	4.0	16.0
CRM_3_15.0w	15.0	4.0	11.0
CRM_3_17.5w	17.5	4.0	13.5
